# Precise lineage tracking using molecular barcodes demonstrates fitness trade-offs for ivermectin resistance in nematodes

**DOI:** 10.1093/g3journal/jkaf081

**Published:** 2025-04-10

**Authors:** Zachary C Stevenson, Eleanor Laufer, Annette O Estevez, Kristin Robinson, Patrick C Phillips

**Affiliations:** Institute of Ecology and Evolution, University of Oregon, Eugene, OR 97401, United States; Institute of Ecology and Evolution, University of Oregon, Eugene, OR 97401, United States; Institute of Ecology and Evolution, University of Oregon, Eugene, OR 97401, United States; Institute of Ecology and Evolution, University of Oregon, Eugene, OR 97401, United States; Institute of Ecology and Evolution, University of Oregon, Eugene, OR 97401, United States

**Keywords:** barcode, barcoded, lineage tracking, ivermectin, fitness, selection, environmentally-dependent selection, WormBase

## Abstract

A fundamental tenet of evolutionary genetics is that the direction and strength of selection on individual loci vary with the environment. Barcoded evolutionary lineage tracking is a powerful approach for high-throughput measurement of selection within experimental evolution that to date has largely been restricted to studies within microbial systems, largely because the random integration of barcodes within animals is limited by physical and molecular protection of the germline. Here, we use the recently developed TARDIS barcoding system in *Caenorhabditis elegans* to implement the first randomly inserted genomic-barcode fitness experiment within an animal model and use this system to precisely measure the influence of the concentration of the anthelmintic compound ivermectin on the strength of selection on an ivermectin resistance cassette. The combination of the trio of knockouts in neuronally expressed GluCl channels, *avr-14*, *avr-15*, and *glc-1*, has been previously demonstrated to provide resistance to ivermectin at high concentrations. Varying the concentration of ivermectin in liquid culture allows the strength of selection on these genes to be precisely controlled within populations of millions of individuals, with the frequency of each barcode then being measured at multiple time points via sequencing at deep coverage and used to estimate the fitness of the individual lineages in the population. The mutations display a high cost to resistance at low concentrations, rapidly losing out to wild-type genotypes, but the balance tips in their favor when the ivermectin concentration exceeds 2 nM. This trade-off in resistance is likely generated by a hindered rate of development in resistant individuals. Our results demonstrate that *C. elegans* can be used to generate high-precision estimates of fitness using a high-throughput barcoding approach to yield novel insights into evolutionarily and economically important traits.

## Introduction

The interplay between environmental context and the direction and strength of selection on individual loci has been the fundamental underpinning of evolutionary genetics since its inception. Most standard population genetic models are grounded in 2 essential features: (1) that selection is relatively constant over time and (2) that selection can be best estimated in an aggregate fashion across all alleles with similar phenotypic outcomes. However, populations are never static—new mutations constantly arise and the environment is ever changing—meaning that the pattern of selection on individual loci is likely to change often, sometimes dramatically so (Bell 2010). If there is a temporal or spatial structure to these environmental differences, then this variation can potentially lead to the maintenance of genetic diversity (Abdul-Rahman *et al.* 2021). Several examples of this process exist for antibiotics, small molecules, and more complex traits such as stress tolerance (Rudman *et al.* 2022). While initial formulations of population genetics focused on allelic change in a cross-sectional, generation-by-generation fashion, over the last several decades developments in molecular population genetics have shifted the focus on the importance of coalescence of evolutionary lineages for full inference of the evolutionary process, usually with a retrospective view (Wakely 2008).

Fitness competition experiments are essential for assessing the relative fitness of different genotypes or populations under controlled conditions. These assays typically involve mixing defined proportions of wild-type and competitor strains, exposing them to specific environmental pressures, and measuring changes in their relative abundances over time. However, such experiments are prone to sampling variation, particularly when working with small population sizes. Insufficient sampling can introduce significant fluctuations in observed fitness differences, potentially obscuring true biological effects (Crombie *et al.* 2018). Additionally, the interpretation of fitness outcomes can be confounded by stochastic effects and genetic drift in small populations. These random processes can disproportionately influence allele frequencies, leading to results that may not accurately reflect underlying fitness differences between genotypes.

To address these challenges, molecular tagging methods—such as the incorporation of fluorescent markers like GFP—have been employed. However, fluorescent markers are limited in number, can impose fitness costs, and may further confound fitness measurements. In contrast, random molecular barcoding, which involves adding small, neutral DNA sequences to the genome, offers an optimal solution for tracking evolutionary lineages in large-scale experimental evolution and fitness competition studies. Random barcoding approaches have been developed for microbial systems, including bacteria and yeast, enabling lineage-based analyses within a fully experimental framework (Blundell and Levy 2014; Levy *et al.* 2015; Jahn *et al.* 2018; Ba *et al.* 2019; Jasinska *et al.* 2020). Barcoding provides a distinct advantage in fitness competition experiments by addressing a key confounding issue: de novo mutations. By randomly barcoding many individuals of the same genotype, outlier lineages within that genotype can be identified and excluded from analysis, improving the rigor and quality of fitness measurements.

Yet, similar barcoding technologies have heretofore been missing for animals, largely because it is very difficult to transduce large libraries of DNA barcodes directly into animal gametes (Stevenson *et al.* 2023). We have recently developed a library-based transgenesis system called Transgenic Arrays Resulting in Diverse Integrated Sequences (TARDIS) within the nematode *Caenorhabditis elegans* that overcomes this barrier in 2 steps: first by creating a diverse barcode library within the individual using an extra chromosomal array and then secondarily randomly incorporating individual barcode elements into a defined landing pad location via CRISPR/Cas9 activation in a subsequent generation (Stevenson *et al.* 2023). Here, we provide an exemplar for the application of TARDIS barcoding within experimental systems by exploring potential trade-offs in natural selection for resistance across a gradient of concentrations of the anthelmintic ivermectin, demonstrating that lineage-based approaches in experimental evolution, and within fitness competition experiments as we present here, can serve as a powerful means of generating high-precision estimates of the magnitude of natural selection in the face of environmental variation.

Ivermectin, the most widely used anthelmintic drug (Gill *et al.* 1991; Campbell 1993; Shoop 1993; Prichard 2007; Leathwick *et al.* 2012; Geurden *et al.* 2015) is used worldwide for controlling nematode parasitic infestations both within livestock and companion animals. In humans, ivermectin is used for the treatment of crippling parasite diseases such as ascariasis, which can infect the lung and intestines, and onchocerciasis, which causes river blindness (Conterno *et al.* 2020; Leung *et al.* 2020; Sulik *et al.* 2023). Rapid development of resistance to ivermectin in particular is a growing problem (Doyle *et al.* 2022). From a border evolutionary perspective, insecticide resistance, which includes ivermectin, has long served as an important exemplar of evolution within natural populations (Crow 1974; Mallet 1989; ffrench-Constant 2013; Freeman *et al.* 2021; Pu and Chung 2024). Ivermectin works by activating the glutamate-gated chloride (GluCl) channels, leading to hyperpolarization (Dent *et al.* 1997; Ardelli *et al.* 2009). Within laboratory populations of *C. elegans*, these neurological effects can be quantified by measuring the rate of muscle-based phenotypes, such as pharyngeal pumping (Weeks *et al.* 2018). Extensive screening efforts have identified 3 mutations in the loci encoding GluCl channels (*avr-14*, *avr-15*, and *glc-1*) that, in combination, lead to a roughly 4000X increase in resistance to ivermectin (Dent *et al.* 2000; Shaver *et al.* 2024). For our competition experiment, we selected to use the strain JD608 (*avr-14*(ad1302)I; *avr-15*(ad1051)*glc-1*(pk54)V) which is mutated for these 3 GluCl genes. The combination of the underlying functional biology of these mutants and the power of *C. elegans* as a system for experiments studying evolutionary parameters in the lab (Teotónio *et al.* 2017) makes this an especially powerful approach to address the question of adaptive mutations for ivermectin resistance in nematodes.

**Fig. 1.**
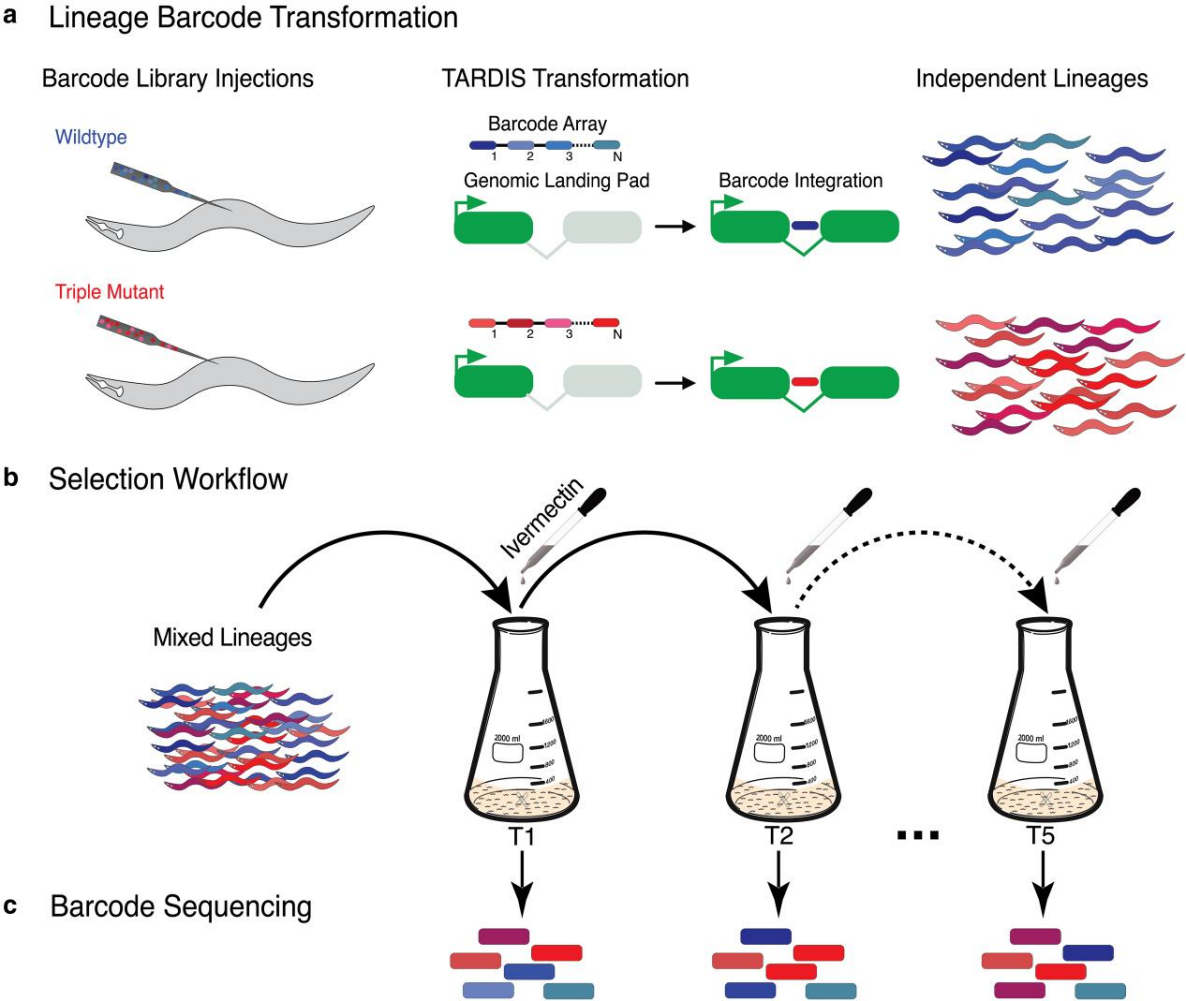
Experimental overview. a) Lineage transformation following TARDIS transgenesis. Barcodes are integrated within synthetic introns for hygromycin B resistance and were engineered to contain “constant” bases to distinguish from which genetic background lineages originated. b) 30–60 lineages were then pooled and serially cultured in various concentrations of ivermectin (0 to 5 nM with 1 nM increments) for a total of 5 transfers (T1–T5). c) At each transfer, a portion of the population was lysed and genomic DNA was extracted. Barcodes were amplified and quantified by Illumina Sequencing.

Here, we report the first-ever randomly barcoded fitness competition experiment performed within an animal system, *Caenorhabditis elegans*, which allows replicated measurements of selection coefficients of a known mutant within a well-defined environmental context. We barcode populations of *C. elegans* utilizing TARDIS—a high-throughput transgenic methodology (Stevenson *et al.* 2023)—with unique collections of barcodes to distinguish between wild-type and triple mutant backgrounds (Fig. 1a). We also present a modified liquid culture protocol for growing several multimillion sized animal populations in parallel (Fig. 1b, Supplementary Fig. 1), making this experiment, to our knowledge, the largest animal-based fitness competition study conducted to date. By sequencing barcodes on each transfer (Fig. 1c), we found that selection is dependent on the concentration of ivermectin and illustrates a clear fitness trade-off depending on concentration. This trade-off manifests phenotypically as a trade-off in developmental rate, which is reduced in the unfavored genetic background. Our project serves as an initial exemplar of lineage tracking utilizing random barcodes within an animal model. We believe the methods can be applied generally to study fitness and lineage dynamics in experimental populations. Broadly, the methods and results we present here, show the potential to perform large-scale experimental evolution within animal systems.

## Methods

### *C. elegans* strain maintenance

Unless otherwise indicated for the liquid culture experimental evolutions, *C. elegans* strains were maintained at 20°C on nematode growth media (NGM) plates seeded with *Escherichia coli*  OP50.

### Generation of barcoded lineages

To create the background triple mutant strain with the TARDIS barcode landing pad, JD608  *avr-14*(ad1302)I; *avr-15*(ad1051)*glc-1*(pk54)V was crossed with PX740  N2-PD1073 fxIs47[*rsp-0p*:: 5′ Δ*HygR*:: GCGAAGTGACGGTAGACCGT:: 3′ Δ*HygR*::*unc-54* 3′::LoxP, II:8420157] II:8420157 to create PX776 *avr-14*(ad1302)I; fxIs47; *avr-15*(ad1051) *glc-1*(pk54)V. PX776 was injected with TARDIS barcodes following the protocols of Stevenson *et al.* (2023), with a unique barcode sequence “NANNNTNTNNCNNNN” to facilitate correct identification of the mutant by sequencing, resulting in PX787 *avr-14*(ad1302)I; *avr-15*(ad1051) *glc-1*(pk54)V; fxEx29 [TARDIS 5′Δ*HygR*::Intron5′::Read1::NANNNTNTNNCNNNN::Read2::Intron3′::3′ Δ*HygR hsp- 16.41p*::*piOptCas9*::*tbb- 2* 3 “UTR + rsp-27p::*NeoR*::*unc-54* 3′ UTR + U6p:: GCGAAGTGACGGTAGACCGT]; fxIs47. For the wild-type barcoded TARDIS array, PX786 was used and described in Stevenson *et al.* (2023). Lineages were generated from both PX786 and PX787 following standard TARDIS-integrated protocols (Stevenson *et al.* 2023). Briefly, TARDIS-array bearing strains were hypochlorite synchronized, and heat shocked at the L1 stage to integrate barcodes, marking the lineage. Several lineages were isolated and identified by Sanger sequencing (Azenta Life Sciences, South Plainfield, NJ, USA).

### Competition experiments, liquid culture, and sample collection

To create our liquid environment, we used NGM buffer as our base (Leung *et al.* 2011), in addition, we added 100 µg/ml carbenicillin, 5 µg/ml cholesterol, 125 µg/ml hygromycin B, and 10 µg/ml nystatin. A 10 µM ivermectin/DMSO stock­ was diluted further with DMSO to achieve the desired experimental molarity. DMSO only was used for all the controls and all (DMSO) (including the controls) were normalized for each experimental set while maintaining a final (DMSO) of ≤1% (AlOkda and Raamsdonk 2022). 4 × 10^9^ PXKR1 cells/ml (NA22 transformed with pUC19 for carbenicillin resistance) were also added (Stevenson *et al.* 2023). Bacteria were grown in several large batches and measured for cell concentration before being frozen at −80°C until needed. Independent lineage populations were started by allowing large density plates (100 mm) to reach starvation and then each lineage was added independently into a liquid solution. Total number of individuals lineages is provided in Supplementary data file 1 for each condition. Lineages were then mixed for the parental generation at ∼10% wild type, 90% mutant for ivermectin concentrations of 0 nM; and 30% wild type, 70% mutant for1 nM; while for 2 , 3, 4, and 5 nM, populations were mixed at approximately equal concentrations. While developing these methods, we observed the point of neutrality at ∼3 nM. To maximize our power to measure the selective advantages of these conditions, we decided to start the parental populations at unequal frequencies, particularly at the lower end where wild type was outcompeting. Additionally, we added more experimental replicate populations at 3 nM, anticipating the point of neutrality would be close to 3 nM and provide additional power in our analysis should that be the case. However, our higher throughput experiments showed this to be closer to ∼1.5 nM. Each parental population was started with several thousand individuals (Supplementary data file 1). Serial cultures were grown in 300 ml volumes in 2L flasks mixed with magnetic stir bars and 10% of the population by volume was transferred every 5 days. Cultures were maintained at a constant 20°C in a temperature-controlled room (Supplementary Fig. 1). Population densities were estimated by counting 6 individual drops ranging from 2 to 20 µl on a glass slide. In some cases, a 10× dilution was made to simplify the counts. Several 1 ml samples were taken on the day of transfer and frozen at −20°C. In cases of lower population densities, 10–50 ml samples were taken and centrifuged to create a pellet to ensure extra genomic DNA could be acquired. Samples were then processed for genomic DNA and barcode frequency as described in Stevenson *et al.* (2023).

### Fitness estimation and analysis of data with FitSeq

Barcode frequencies derived from Illumina sequencing (see Stevenson *et al.* 2023) were provided to PyFitSeq–a python implementation of FitSeq (Li *et al.* 2018). Briefly, FitSeq requires the user to provide the approximate generation time per transfer, which was approximately 1 generation per transfer, along with estimated population sizes. Only mutant lineages which survived to the end of the experiment–fitness greater than −1– were counted. Mutant lineages counts with 10 or less were excluded from the analysis. Mutant selection coefficients were normalized to the average wild-type fitness. Barcodes which did not confirm to the following sequence “NANNNTNTNNCNNNN” for mutant and “NNNCNNTNTNANNN” for wild type were errors due to either sequencing or preparation of sequencing and excluded from the analysis. Raw data and exact counts can be found within the Supplementary data file 1.

### Probability of reaching adulthood during ivermectin exposure

Individual barcoded lineages PX905 (wild-type background) fxSi67 [*rsp-0p*:: 3′ Δ *HygR*::Intron5′::Read1::GAGCAATTTAATCAT::Read2::Intron3′::*unc-54* 3′::LoxP, II:8420157] and PX925 (mutant background) avr-14(ad1302)I; fxSi66 [*rsp-0p*:: 3′ Δ *HygR*::Intron5′::Read1::GATAATATACCTAGT::Read2::Intron3′::*unc-54* 3′::LoxP, II:8420157]; *avr-15*(ad1051*) glc-1*(pk54)V were grown on plates until the population contained mostly gravid adults. The populations were then synchronized in NGM buffer by bleaching the adults in a solution of 1% sodium hypochlorite/0.5% NaOH to collect the eggs. For each strain, eggs were counted postsynchronization (as described above), and liquid culture solutions were made which contained 1 egg/µl. Cultures were then exposed to DMSO or DMSO with concentrations of ivermectin in a liquid culture solution as described above, except they were grown in 15 ml conical tubes and allowed to rotate at 20°C to ensure proper mixing and aeration. Total liquid volumes were 5 ml for each culture to allow substantial air space within the tubes. Just prior to counting, populations were immobilized with 0.2 mM levamisole. For each timepoint, several 20 µl drops were scored for both the total number of animals and the number of animals that had reached the adult stage at 2 separate timepoints, 72 and 96 h postsynchronization, to obtain the percentage adults. Animals were determined to be adults if gravid (eggs observed) or if no eggs, by examining individual animals for mature vulva development at the highest magnification (112.5×).

### Microscopy

The 600 µl samples from the developmental liquid cultures were centrifuged and 550 µl of the supernatant was removed to create a denser population for imaging (∼50 µl). The 3 µl of each worm concentrate was then placed onto a glass slide and cover slipped. Imaging was performed on an Olympus IX73 using cellSens software v2.3. Samples were imaged under white light for 20 ms exposures using a 4× objective. Scale bars were added using Fiji (imageJ) v2.9.0/1.53t.

### Software and statistical analysis

Lineage frequencies were visualized with matplotlib 3.5.2 and data was analyzed with Python 3.7.13. For selection coefficient, peak census size, and developmental trade-offs, plots and statistics were generated in R v.4.3.2 (R Core Team 2023), lmer version v.1.1.35.3 (Bates *et al.* 2015), and visualized using ggplot2 v.3.5.1 (Wickham 2016). Because we have nested replicates, we used a least means squared approach, which was calculated using the emmeans package v. 1.10.1. All calculated values present in Supplementary data file 1. All code was executed in either Jupyter Notebooks v3.6.3 (Google Colab)–stacked frequency plots, or Jupyter Labs v7.9.0 (Kluyver *et al.* 2016)—all statistics done in R, along with plots for the selection coefficients and developmental trajectories.

## Results

### Ivermectin exposure creates environmentally dependent and dynamic selection across generations

**Fig. 2.**
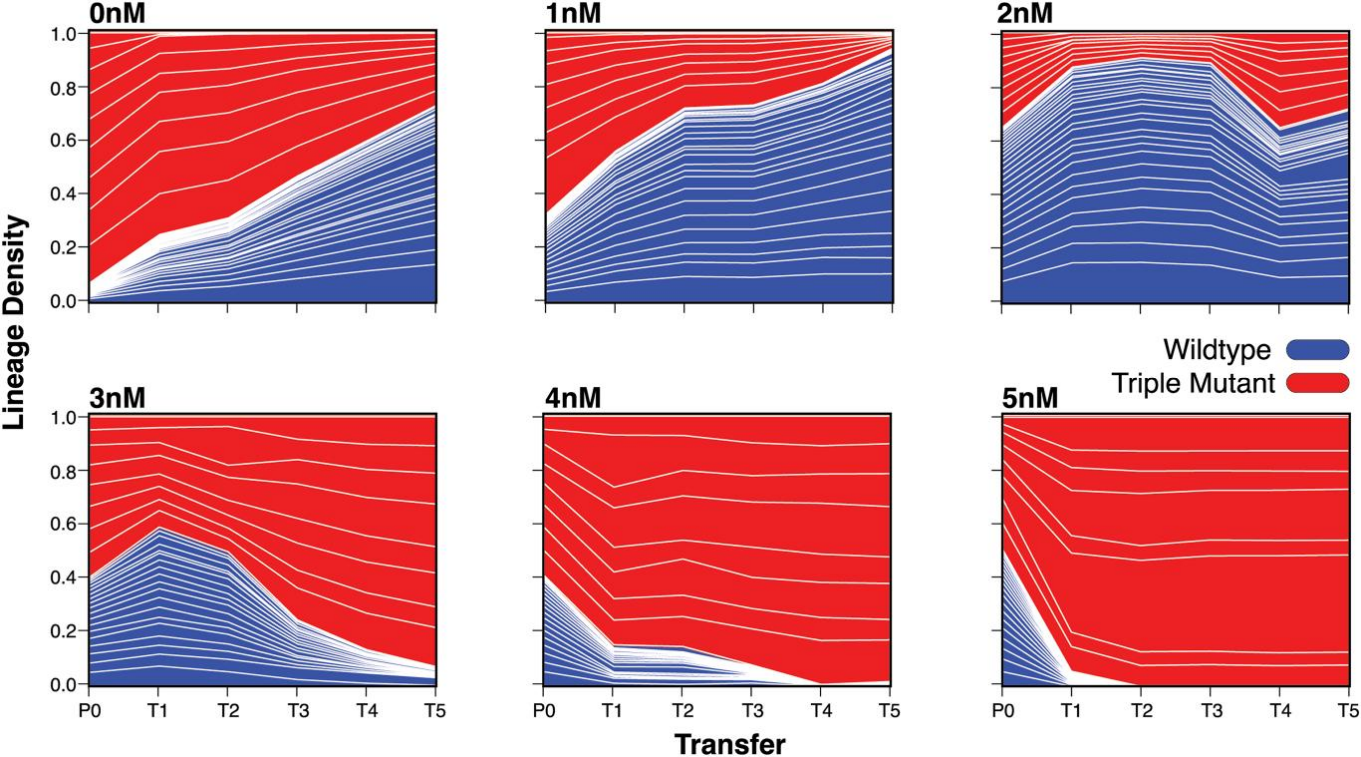
Example lineage frequencies across ivermectin conditions. Transfers (T1–T5) are denoted along the *x*-axis with the parental generation (P0). Lineage density as measured by the barcode frequency is denoted on the *y*-axis. At P0, wild-type (blue) and triple mutant (red) lineages were combined at different starting frequencies that varied with (ivermectin). For 0 and 1 nM we see a clear trend toward a wild-type advantage–there is a cost for being resistant to ivermectin. In the 2 nM condition we start to see the wild-type lineage receiving less of an advantage. For 3, 4, and 5 nM we see a trend toward increasing the triple mutant frequency for each condition.

We performed a competition experiment with multiple barcoded lineages of both wild type and mutant backgrounds in large scale liquid culture and several concentrations of ivermectin exposure. In this way, the mutations and wild-type individuals are “identical by kind” as determined by their allelic state, but only individuals within a given barcoded lineage are “identical by descent” (Lewontin 1986). Notably however, the relatedness within each genotype-class is closer to one another given their individual TARDIS array backgrounds. Cultures were serially transferred a total of 5 times (T1–T5) with barcode frequencies measured at each transfer (Fig. 2), allowing us to access the density of each lineage within the populations. Census size populations were generally maintained above 10^5^ and often surpassed 10^6^ individuals (Supplementary Fig. 2). With transfer population sizes at 10% of the census size, our experiments were designed to minimize the influence of drift in conditions where selection was strong (e.g. 0 and ≥3 nM). However, near the inferred crossover point (∼1.5 nM), where selection coefficients approach neutrality, drift may still contribute to allele frequency dynamics. Overall, we observed a gradient of selection overtime, with lower (ivermectin) favoring the wild-type and higher concentrations favoring the triple mutant (Fig. 2).

**Fig. 3.**
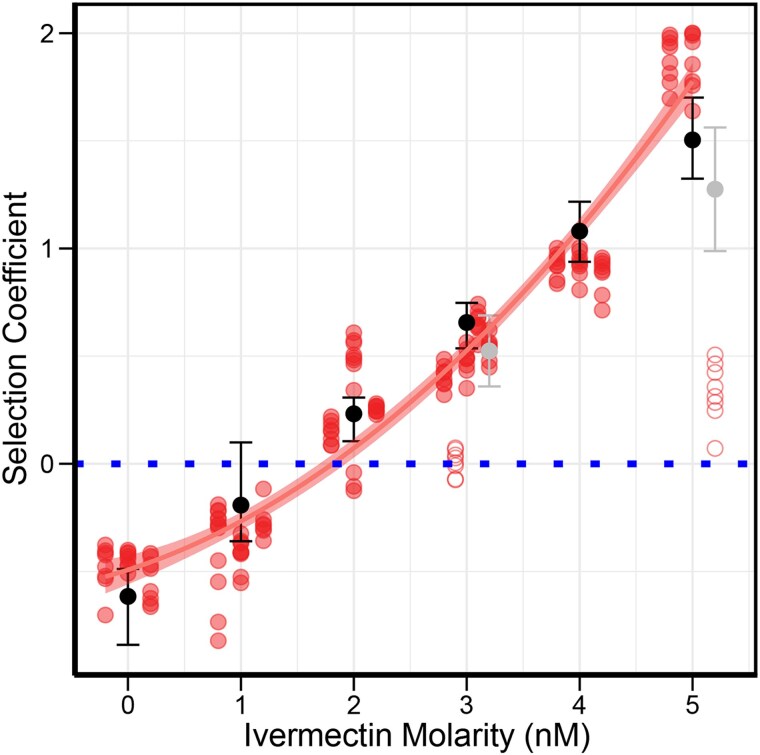
Triple mutant selection coefficient in relation to ivermectin concentration. Blue dotted line represents the wild-type selection coefficient, which was normalized to zero for each concentration. Each selection condition has 3 replicates, while 3 nM has an additional 2 replicates. Individual replicates are jittered into columns within an ivermectin concentration. Hollow circles represent outlier replicates circles that were excluded from curve fitting. Black bars represent the least mean squared confidence intervals for each concentration. Gray bars are also the least means squared confidence interval include the outlier replicates. Fitted polynomial is *S* = 0.053(ivermectin)^2^ + 0.18(ivermectin)−0.5, adjusted *R*^2^ = 0.94, *P*-value=<2.2 × 10^−16^. We see a clear exponential increase in fitness with concentration for the triple mutant lineages.

In our control (Fig. 2, 0 nM), the wild-type background was favored suggesting that without selection pressure there was a significant deleterious cost associated with the triple mutant background. At the lowest concentrations of ivermectin (Fig. 2, 1 nM), the advantage of the wild-type background remains, however, it is lessened in comparison to the 0 nM condition. With increasing concentrations of ivermectin, a transition around the 2 nM concentration occurs whereby the triple mutant becomes increasingly favored with each stepwise increase in [ivermectin] (Fig. 2, 3–5 nM). Interestingly, selection was not constant and, in some cases, shifted from transfer to transfer (see Fig. 2, 1 nM T2 to T3 and 2 nM T4 to T5). This variability, combined with the early wild-type advantage in some cases (Fig. 2, 2 and 3 nM, P0 to T1), clearly suggests that multiple generations should be used to accurately measure fitness when performing competition experiments. Among our lineages, we clearly saw each genetically-identical lineage following the same selective trajectory. Having multiple lineages within the same genetic background provided an internal quality control, which increases our confidence in the measurement of selection, since an adaptive mutation of large effect within the background would normally be invisible in standard competition experiments.

### Mutant selection coefficients increase with ivermectin concentration

Utilizing the barcode frequencies, we were able to estimate the selection coefficients of each lineage from the mixed populations after the final transfer (T5). Each lineage acted as a separate measurement of the strength of selection and provided internal replication and measured variation in the selection coefficient for that population. With the wild-type selection coefficients held constant at zero, selection on the triple mutant rose exponentially alongside [ivermectin] with a strong correlation (adjusted *R*^2^ = 0.94, *F*_2,149_, *P* < 2.2 × 10^−16^) (Fig. 3). Without ivermectin (0 nM), the triple mutant was conditionally deleterious with a selection coefficient of approximately *S* ≈ −0.5. Thus, there was a clear trade-off conferred in the absence of ivermectin. By simply increasing the ivermectin concentration, we were able to lessen the selective pressure and swap the deleterious and advantaged backgrounds. Similar to our lineage trajectories, the wild-type genotype was favored under 2 nM, with a point of neutrality at ∼1.5 nM. Generally, we observed large shifts in the selection coefficient per condition. Additional transfers were essential for accurately estimating the selection coefficients, as we generally saw fluctuations in the transfer early on (Fig. 2). With additional, intermediate, concentrations of ivermectin, and larger number of transfers, even finer resolution of selection coefficients could possibly be achieved. We observed 2 outlier replicates, 1 in 3 nM and 1 in 5 nM conditions in which the grouping of triple mutant lineages presented a much lower selection coefficients than the other replicate conditions. While it is unclear why they are outliers (as discussed below), the overall trends remain unchanged.

### Ivermectin resistance generates a trade-off on developmental rate

**Fig. 4.**
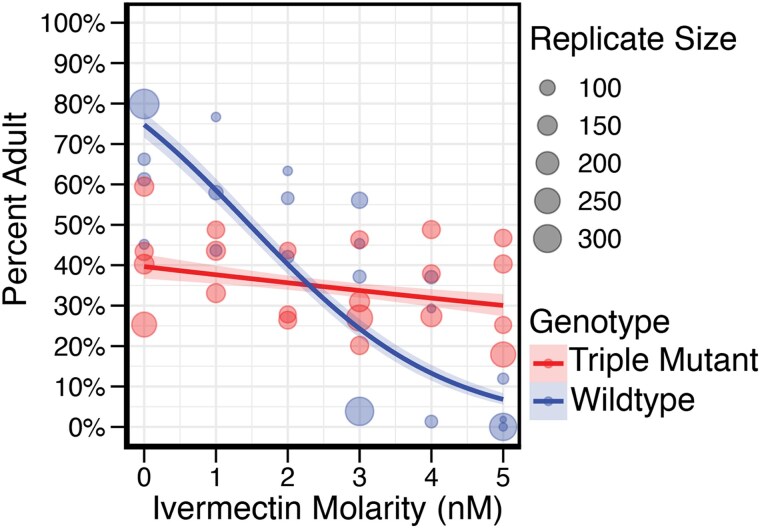
Developmental delay at 72 h while developing in ivermectin. Ratios represent the number of adults over the total number counted. Wild type is represented in blue, while the triple mutant is represented in red. Total worms counted per replicate is represented by circle size. We see a clear impact of ivermectin on development (*P*-value=<2.2 × 10^−16^). Shading denotes 95% confidence interval. We see the wild-type background is more advanced in development compared to the triple mutant background at the 0 nM concentration up until approximately 3 nM, where the triple mutant shows a developmental advantage. At all 5 conditions we see the triple mutant background is developing at relatively the same rate.

While maintaining JD608 (triple GluCl mutant background) and N2 (WT) strains, we observed a noticeable delay in development for the triple mutant strain compared to wild type in the absence of ivermectin. In contrast, when grown on plates in the presence of ivermectin, we observed that wild-type worms were significantly delayed, leading us to hypothesize that delayed development, and a concomitant delay in reproduction, could be the source of selective tradeoff to ivermectin. To test our hypothesis, we hypochlorite synchronized a single barcoded lineage from both the wild-type and triple mutant backgrounds and exposed them to ivermectin in a liquid culture environment that paralleled the conditions that we used for the selection experiment. At 2 separate time points (72 and 96 h postsynchronization), we took samples from the culture and counted the total number of worms, and what proportion had reached adulthood (Fig. 4, 72 h postsynchronization, Supplementary Fig. 3, 96 hours post-synchronization). We found there to be a strong correlation with reduced developmental rate and ivermectin concentration (72 h, *P* < 2.2 × 10^−16^). At 0 nM, the development of the animals with the wild-type background outpaced the development of those with the triple mutant background. This advanced development likely provides a significant adaptive advantage to the animals with the wild-type background when compared to the triple mutant animals competing in the same environment. However, when animals are grown in increasing ivermectin concentrations, wild-type development is “stunted.”

Development of the wild-type background animals when grown in 3 nM ivermectin appears to be less synchronous, with distribution across several larval stages, and adults making up a significantly smaller proportion of the total progeny numbers when compared to those grown without ivermectin. Additionally, when compared to the triple mutant background animals grown in the presence of 3 nM ivermectin, there was no significant difference in the percentage of adults between the 2 strains. At our highest concentration of ivermectin, 5 nM, we observed very few adults from the wild-type background animals (3.4% compared to the 63.1% observed at 0 nM, 72 h). In contrast, the triple mutant background animals developed at approximately the same rate across all ivermectin concentrations although there was a slight initial delay at 72 h that was not observed after 96 h (Fig. 4 compared to Supplementary Fig. 3). Overall, then, the developmental delay hypothesis is strongly supported, with a clear trend toward slower development for the wild-type strain on increasing concentrations of ivermectin. Remarkably, we observe a similar crossover point for the selection experiment and the development. With the selection crossover approximately at 1.5 nM, and ∼2.5 nM for the development.

## Discussion

Darwin (1859) is most recognized for introducing the idea of natural selection, but it is really his overall vision of “descent with modification” that captures the entire scope of the evolutionary process. Full reconciliation of phylogenetic, molecular evolutionary, and population genetic perspectives on evolutionary relationships depends on being able to tie microevolutionary processes to lineage-based estimates of evolutionary change. Here we present the first randomly barcoded fitness competition experiment performed within an animal system. Our results demonstrate that we can precisely and reproducibly measure selection within a specific environmental context and change the evolutionary advantage of a given haplotype by changing the environment in which it is found. Utilizing our liquid culture approach with the nematicide ivermectin as the selective agent, we were able to grow populations in the many millions, creating the largest fitness competition conducted within an animal system to date. The application of our new unique random barcoding system used here both provides interesting insights into an important agricultural intervention and paves the way for the application of this technology to a wide set of important evolutionary questions.

### Naturally occurring genetic variation and hypothesis testing of ivermectin resistance

Resistance to ivermectin has already been widely observed within natural populations of nematodes (Hawkins *et al.* 2019). Likely, as the application of ivermectin cannot be maintained at a constant level, parasites are exposed to below-therapeutic concentrations of ivermectin in either missed doses or incomplete treatments, providing an opportunity for ivermectin-resistant mutations to increase in frequency in the population before they can be eliminated via a lethal dose (Fissiha and Kinde 2021). We used a system of 3 synthetic resistance mutations to establish the framework for fitness competitions and barcode lineage tracking used here. Specifically, we selected mutations known for strong resistance to ivermectin (Dent *et al.* 2000). While this system is certainly artificial to a degree, it is possible to create “parasitized” strains of *C. elegans* in which the native allele is swapped for a resistance allele from a natural parasite could provide further insight into the evolution of anthelmintic resistance utilizing a non-parasitic lab model (Zamanian and Andersen 2016). Within natural nematode populations, potential resistance alleles in *avr-14* and *avr-15* have yet to be found (Doyle *et al.* 2022), however there is evidence for selective mutations within *glc-1* (Ghosh *et al.* 2012), as well as in other *C. elegans* homologs such as the *cky-1* mutation found in the parasitic nematode *Haemonchus contortus* (Doyle *et al.* 2022). There is also evidence of other potential ivermectin resistance genes within *C. elegans* whose effects could be quantified more precisely using the methods developed here (Su and Dent 2015). Creating “synthetic” version of natural alleles within model nematode species such as *C. elegans* opens the potential to ask hypothesis about natural variation and fitness, while contained within the controls of a lab environment. Within the lab, and utilizing protocols we outline in this manuscript, it is possible to measure minor changes in evolutionary fitness that may be impossible to identify simply by observing natural allele frequencies.

We used all 3 mutations in tandem for this study, but because we were able to measure selection in the triple mutant background, it should be possible to barcode individual strains with single or double mutations in *avr-14*, *avr-15*, and *glc-1* to discover possible adaptive intermediates across a range of concentrations. Shaver *et al.* (2024) have recently competed individual strains with new knockout alleles for *avr-14*, *avr-15*, and *glc-1*, which are not the canonical alleles used in our study. While qualitatively our studies are similarly aligned, the magnitude of selection is different. This is likely due to the environment (plate vs liquid) and the specifics in terms of measuring selection every few generations in Shaver *et al.*, compared to ours being approximately a single generation. The specific contribution of functional interactions among these genes to the pattern of natural selection we observe here certainly merits further investigation.

### Pleiotropic effects and trade-offs in adaptation

Pleiotropic effects of potentially beneficial mutations are thought to be widespread within genetic systems (Zhang 2023). The consequences of pleiotropic effects can lead to adaptive trade-offs, where a mutation can provide a benefit within 1 environmental context and not within another (Roff 1992; Giannattasio *et al.* 2013; Wang *et al.* 2015; Jerison *et al.* 2020; Bakerlee *et al.* 2021; Schmidlin *et al.* 2024). A similar example of an adaptive trade-off occurs between the garter snake, *Thamnophis sirtalis*, and its prey, the newt *Taricha granulosa,* which produces a neurotoxin, tetrodoxin (TTX) as an antipredator defense (Brodie III and Brodie Jr., 1990, 1991, 1999). While resistance to TTX has evolved in the garter snake it is also accompanied by an adaptive trade-off in which garter snakes with resistant-mutations in the TTX-binding site of Nav1.4, a voltage gated-sodium channel, have impaired movement (del Carlo *et al.* 2024). A physiological tradeoff with resistance at the level of neuronal signaling of this kind appears to mirror the results seen here, in which we find a clear adaptive trade-off between ivermectin resistance and developmental rate in our experimental populations. A trade-off of this kind could potentially help drive the dynamics of natural resistance to ivermectin in the field, and indeed, when exposed to increasing concentrations of ivermectin the parasitic nematode *Haemonchus contortus* shows decreasing rates of larval development (Tuersong *et al.* 2022). While it is not known if resistance to ivermectin generally comes at a cost to development, given our results, it is reasonable to hypothesize natural population could be found that exhibit this trade-off. Experimental evolution in the laboratory, where selective and environmental conditions can be finely controlled, can be used to develop precise hypotheses that can be tested within natural populations where the complexity of mitigating factors might often confound our ability to cleanly addressing a specific functional hypothesis.

### Advantages and applications of multilineage barcoding in assessing mutant fitness and evolutionary dynamics

Strictly speaking, our random barcoding approach for fitness competitions is not absolutely necessary, as it is possible to assess mutant allele frequencies directly, or by using a simple co-marker such as fluorescence or molecular probes (Murray and Cutter 2011; Kasimatis *et al.* 2022; Webster *et al.* 2022; Shaver *et al.* 2024). There are several distinct advantages to testing multilineage barcodes of the same allelic set, however. First, each lineage provides a replicated estimate of fitness in a given environment and trial. This allows a level of precision and statistical rigor that would otherwise be impossible. In our case, we found that lineage-specific fitness estimates tended to be very similar and to provide excellent estimates of triple mutant fitness. The interesting exception is when the concentration of ivermectin was right at the trade-off balance point (∼2 nM). Here we saw a substantial increase in among-lineage variance within each replicate, as might be expected when a haplotype is near the neutral threshold. Within our experiment, replicated lineage estimates were essential for providing high precision estimates of fitness, particularly when the selection coefficient was near zero. We also observed a few replicates in which the ivermectin addition and/or response were clear outliers (e.g. a single replicate in both 3 and 5 nM both showed large deviations from the other replicates). Yet, since all the lineages responded in the same aberrant way—in a manner that was completely inconsistent with the entire experiment—we felt confident that the entire replicate had an unknown error during the execution of the experiment (possible causes could be accidental misapplication of ivermectin, a contaminate in the flask which persisted across the replicate, or microenvironmental changes which could impact the overall response to ivermectin) rather than being part of “normal” sampling variance across genotypes. However, it is important to note that our overall conclusions are completely unchanged if these replicates are included in the analysis, as only minor quantitative details of the response function are altered (Fig. 3). Second, having replicated lineages protects against a very serious confounding factor in multigeneration and large population fitness competitions: de novo background mutations. For example, if a new “high fitness” mutation arises spontaneously during the course of the experiment, it is impossible to separate its effect from the main effect of the triple mutant which is under study, especially when only assessing the allele frequency of the triple mutant itself. While we did not observe this in our current study, we have anecdotally observed this phenomenon while perfecting these methods, and it most certainly would be a caveat for experiments that run for longer durations than those presented here. During preliminary experiments, we observed divergent lineages after 10 transfers (twice as long as presented in this study) in 2 separate replicates.

While this project implements the analysis of randomly barcoded lineages within an animal system for the first time, microbial systems using similar approaches have been well developed for evolutionary studies, particularly for estimating the distribution of fitness effects in large experimental evolution studies (Blundell and Levy 2014; Levy *et al.* 2015; Ba *et al.* 2019). The ability to link novel mutations to unique lineages—and therefore unique evolutionary histories—is of course the real strength of barcoding based approaches. Our work establishes the groundwork for being able to conduct similar experiments in intact multicellular animals. Similar to microbial systems, we capitalized upon the self-fertilizing nature of hermaphroditic *C. elegans* to “lock” a barcode within a lineage, mimicking asexual reproduction used in microbial lineage tracking experiments. However, barcoding under sexual reproduction could be feasible by barcoding across multiple haplotypes of a chromosome and could be applied to a variety of questions regarding sexual selection, sexual dimorphism, and adaptation (Kasimatis *et al.* 2021).

Our current study, while providing important insights into fitness trade-offs, also demonstrates that this barcoding can be used more generally. In establishing the TARDIS system, we showed that it is possible to generate several thousand barcodes via a single injection in a carrier that is a precursor to later lineage work (Stevenson *et al.* 2023), comparable to even the largest barcoded experiments performed in microbes, opening the possibility of performing true evolutionary competition experiments within animal for the first time. While we present a fitness competition experiment here focusing on 2 genotypes, a fully randomized barcoding approach–built upon the TARDIS system–can for the first time also unlock microbial style experimental evolution within an animal system. While we utilized barcodes within our TARDIS system, the potential to study synthetic libraries within animals is also possible, opening the path to variant-competition evolutionary studies similar to microbes (Sharon *et al.* 2018). Combining a number of these precursors together, before barcode integration, TARDIS can be scaled up into the hundreds of thousands of barcodes needed for more general de novo mutation studies broadly. So, in this way, this work points the way toward large scale experimental evolution using de novo mutations within multicellular animals.

## Conclusions

In conclusion, we have presented the first barcoded lineage tracking animal fitness competition, in what is also the largest study of its kind conducted to date. We created a simple experimental design to quantitatively measure selective contributions within a highly controlled environmental context and showed we can experimentally modulate the strength of selection, even changing the adaptive background, by changing the concentration of a simple small molecule drug ivermectin. We find there is an fitness cost to being resistant to ivermectin, which phenotypically manifests in delayed development in the absence of ivermectin. However, in the presence of ivermectin, we find sensitive individuals have highly stunted development, and therefore selection favors the resistant individuals. Our results thus highlight the kind of pleiotropic tradeoff that underlies many central ideas in evolutionary genetics, including the response of natural populations to human interventions such as insecticides and antibiotics.

## Supplementary Material

jkaf081_Supplementary_Data

## Data Availability

The authors affirm that all data necessary for confirming the conclusions of the article are present within the article, figures and tables. All plasmids have been described prior in Stevenson *et al.* (2023). Plasmids pZCS36 (Addgene ID 193048), pZCS41 (Addgene ID 193050) are available through addgene and can be freely viewed with ApE (Davis and Jorgensen 2022). Strains are available upon request. Illumina sequencing data is available at NCBI BioProject ID: PRJNA1170954. All barcoding count information, adult counts, and fitness data are available in Supplementary data file 1. All statistical analysis and code are available in Supplementary data files 2 and 3. Key reagents and strains are available in Supplementary data file 4. Supplemental material available at G3 online.

## References

[jkaf081-B1] Abdul-Rahman F, Tranchina D, Gresham D. 2021. Fluctuating environments maintain genetic diversity through neutral fitness effects and balancing selection. Mol Biol Evol. 38(10):msab173. doi:10.1093/molbev/msab173. PMC8476146 34132791

[jkaf081-B2] AlOkda A, Raamsdonk JMV. 2022. Effect of DMSO on lifespan and physiology in *C. elegans* : implications for use of DMSO as a solvent for compound delivery. microPublication Biol. 2022. doi:10.17912/micropub.biology.000634. PMC9494168 36158529

[jkaf081-B3] Ardelli BF, Stitt LE, Tompkins JB, Prichard RK. 2009. A comparison of the effects of ivermectin and moxidectin on the nematode *Caenorhabditis elegans*. Vet Parasitol. 165(1–2):96–108. doi:10.1016/j.vetpar.2009.06.043. 19631471

[jkaf081-B4] Ba ANN, Cvijović I, Echenique JIR, Lawrence KR, Rego-Costa A, Liu X, Levy SF, Desai MM. 2019. High-resolution lineage tracking reveals travelling wave of adaptation in laboratory yeast. Nature. 575(7783):494–499. doi:10.1038/s41586-019-1749-3. 31723263 PMC6938260

[jkaf081-B5] Bakerlee CW, Phillips AM, Ba ANN, Desai MM. 2021. Dynamics and variability in the pleiotropic effects of adaptation in laboratory budding yeast populations. eLife. 10:e70918. doi:10.7554/elife.70918. 34596043 PMC8579951

[jkaf081-B6] Bates D, Mächler M, Bolker B, Walker S. 2015. Fitting linear mixed-effects models using lme4. J Stat Softw. 67(1):1–48. doi:10.18637/jss.v067.i01.

[jkaf081-B7] Bell G . 2010. Fluctuating selection: the perpetual renewal of adaptation in variable environments. Philos Trans R Soc B: Biol Sci. 365(1537):87–97. doi:10.1098/rstb.2009.0150. PMC2842698 20008388

[jkaf081-B8] Blundell JR, Levy SF. 2014. Beyond genome sequencing: lineage tracking with barcodes to study the dynamics of evolution, infection, and cancer. Genomics. 104(6):417–430. doi:10.1016/j.ygeno.2014.09.005. 25260907

[jkaf081-B9] Brodie ED III, Brodie ED Jr. 1990. Tetrodotoxin resistance in garter snakes: an evolutionary response of predators to dangerous prey. Evolution 44(3):651–659. doi:10.1111/j.1558-5646.1990.tb05945.x. 28567972

[jkaf081-B10] Brodie ED III, Brodie ED Jr. 1991. Evolutionary response of predators to dangerous prey-reduction of toxicity of newts and resistance of garter snakes in island populations. Evolution 45(1):221–224. doi:10.1111/j.1558-5646.1991.tb05280.x. 28564068

[jkaf081-B11] Brodie ED III, Brodie ED Jr. 1999. Costs of exploiting poisonous prey: evolutionary trade-offs in a predator-prey arms race. Evolution 53(2):626. doi:10.2307/2640799. 28565425

[jkaf081-B12] Campbell WC . 1993. Ivermectin, an antiparasitic agent. Med Res Rev. 13(1):61–79. doi:10.1002/med.2610130103. 8416263

[jkaf081-B13] Conterno LO, Turchi MD, Corrêa I, de Barros Almeida RAM. 2020. Anthelmintic drugs for treating ascariasis. Cochrane Database Syst Rev. 4(4):CD010599. doi:10.1002/14651858.cd010599.pub2. 32289194 PMC7156140

[jkaf081-B14] Crombie TA, Saber S, Saxena AS, Egan R, Baer CF. 2018. Head-to-head comparison of three experimental methods of quantifying competitive fitness in *C. elegans*. PLoS One. 13(10):e0201507. doi:10.1371/journal.pone.0201507. 30339672 PMC6195253

[jkaf081-B15] Crow JF . 1974. Genetics of insecticide resistance: general considerations. In: Research Progress on Insect Resistance. Vol. 2. Entomological Society of America. p. 69–74. doi:10.4182/ecem7264.ii-1.69.

[jkaf081-B16] Darwin C . 1859. On the Origin of species by Means of Natural Selection, or the Preservation of Favoured Races in the Struggle for Life. London: J.Murray. PMC5184128 30164232

[jkaf081-B17] Davis MW, Jorgensen EM. 2022. Ape, a plasmid editor: a freely available DNA manipulation and visualization program. Front Bioinform. 2:818619. doi:10.3389/fbinf.2022.818619. 36304290 PMC9580900

[jkaf081-B18] del Carlo RE, Reimche JS, Moniz HA, Hague MTJ, Agarwal SR, Brodie ED, Brodie ED, Leblanc N, Feldman CR. 2024. Coevolution with toxic prey produces functional trade-offs in sodium channels of predatory snakes. eLife. 13:RP94633. doi:10.7554/eLife.94633.1.

[jkaf081-B19] Dent JA, Davis MW, Avery L. 1997. *Avr-15* encodes a chloride channel subunit that mediates inhibitory glutamatergic neurotransmission and ivermectin sensitivity in *Caenorhabditis elegans*. EMBO J. 16(19):5867–5879. doi:10.1093/emboj/16.19.5867. 9312045 PMC1170218

[jkaf081-B20] Dent JA, Smith MM, Vassilatis DK, Avery L. 2000. The genetics of ivermectin resistance in *Caenorhabditis elegans*. Proc Natl Acad Sci U S A. 97(6):2674–2679. doi:10.1073/pnas.97.6.2674. 10716995 PMC15988

[jkaf081-B21] Doyle SR, Laing R, Bartley D, Morrison A, Holroyd N, Maitland K, Antonopoulos A, Chaudhry U, Flis I, Howell S, et al 2022. Genomic landscape of drug response reveals mediators of anthelmintic resistance. Cell Rep. 41(3):111522. doi:10.1016/j.celrep.2022.111522. 36261007 PMC9597552

[jkaf081-B22] ffrench-Constant RH . 2013. The molecular genetics of insecticide resistance. Genetics. 194(4):807–815. doi:10.1534/genetics.112.141895. 23908373 PMC3730913

[jkaf081-B23] Fissiha W, Kinde MZ. 2021. Anthelmintic resistance and its mechanism: a review. Infect Drug Resist. 14:5403–5410. doi:10.2147/idr.s332378. 34938088 PMC8687516

[jkaf081-B24] Freeman JC, Smith LB, Silva JJ, Fan Y, Sun H, Scott JG. 2021. Fitness studies of insecticide resistant strains: lessons learned and future directions. Pest Manag Sci. 77(9):3847–3856. doi:10.1002/ps.6306. 33506993

[jkaf081-B25] Geurden T, Chartier C, Fanke J, di Regalbono AF, Traversa D, von Samson-Himmelstjerna G, Demeler J, Vanimisetti HB, Bartram DJ, Denwood MJ. 2015. Anthelmintic resistance to ivermectin and moxidectin in gastrointestinal nematodes of cattle in Europe. Int J Parasitol Drugs Drug Resist. 5(3):163–171. doi:10.1016/j.ijpddr.2015.08.001. 26448902 PMC4572401

[jkaf081-B26] Ghosh R, Andersen EC, Shapiro JA, Gerke JP, Kruglyak L. 2012. Natural variation in a chloride channel subunit confers avermectin resistance in *C. elegans*. Science. 335(6068):574–578. doi:10.1126/science.1214318. 22301316 PMC3273849

[jkaf081-B27] Giannattasio S, Guaragnella N, Ždralević M, Marra E. 2013. Molecular mechanisms of *Saccharomyces cerevisiae* stress adaptation and programmed cell death in response to acetic acid. Front Microbiol. 4:33. doi:10.3389/fmicb.2013.00033. 23430312 PMC3576806

[jkaf081-B28] Gill JH, Redwin JM, Wyk JAV, Lacey E. 1991. Detection of resistance to ivermectin in *Haemonchus contortus*. Int J Parasitol. 21(7):771–776. doi:10.1016/0020-7519(91)90144-v. 1774112

[jkaf081-B29] Hawkins NJ, Bass C, Dixon A, Neve P. 2019. The evolutionary origins of pesticide resistance. Biol Rev Camb Philos Soc. 94(1):135–155. doi:10.1111/brv.12440. 29971903 PMC6378405

[jkaf081-B30] Jahn LJ, Porse A, Munck C, Simon D, Volkova S, Sommer MOA. 2018. Chromosomal barcoding as a tool for multiplexed phenotypic characterization of laboratory evolved lineages. Sci Rep. 8(1):6961. doi:10.1038/s41598-018-25201-5. 29725068 PMC5934437

[jkaf081-B31] Jasinska W, Manhart M, Lerner J, Gauthier L, Serohijos AWR, Bershtein S. 2020. Chromosomal barcoding of *E. coli* populations reveals lineage diversity dynamics at high resolution. Nat Ecol Evol. 4(3):437–452. doi:10.1038/s41559-020-1103-z. 32094541

[jkaf081-B32] Jerison ER, Ba ANN, Desai MM, Kryazhimskiy S. 2020. Chance and necessity in the pleiotropic consequences of adaptation for budding yeast. Nat Ecol Evol. 4(4):601–611. doi:10.1038/s41559-020-1128-3. 32152531 PMC8063891

[jkaf081-B33] Kasimatis KR, Moerdyk-Schauwecker MJ, Lancaster R, Smith A, Willis JH, Phillips PC. 2022. Post-insemination selection dominates pre-insemination selection in driving rapid evolution of male competitive ability. PLoS Genet. 18(2):e1010063. doi:10.1371/journal.pgen.1010063. 35157717 PMC8880957

[jkaf081-B34] Kasimatis KR, Sánchez-Ramírez S, Stevenson ZC. 2021. Sexual dimorphism through the lens of genome manipulation, forward genetics, and spatiotemporal sequencing. Genome Biol Evol. 13(2):evaa243. doi:10.1093/gbe/evaa243. 33587127 PMC7883666

[jkaf081-B35] Kluyver T, Ragan-Kelley B, Pérez F, Granger B, Bussonnier M, Frederic J, Kelley K, Hamrick J, Grout J, Corlay S, et al 2016. Jupyter Notebooks – a publishing format for reproducible computational workflows. In: Proceedings of the 20th International Conference on Electronic Publishing. p. 87–90.

[jkaf081-B36] Leathwick DM, Waghorn TS, Miller CM, Candy PM, Oliver A-MB. 2012. Managing anthelmintic resistance—use of a combination anthelmintic and leaving some lambs untreated to slow the development of resistance to ivermectin. Vet Parasitol. 187(1–2):285–294. doi:10.1016/j.vetpar.2011.12.021. 22244532

[jkaf081-B38] Leung AKC, Leung AAM, Wong AHC, Hon KL. 2020. Human ascariasis: an updated review. Recent Pat Inflamm Allergy Drug Discov. 14(2):133–145. doi:10.2174/1872213X14666200705235757. 32628606

[jkaf081-B37] Leung CK, Deonarine A, Strange K, Choe KP. 2011. High-throughput screening and biosensing with fluorescent *C. elegans* strains. J Vis Exp. (51):e2745. doi:10.3791/2745. PMC3339844 21633332

[jkaf081-B39] Levy SF, Blundell JR, Venkataram S, Petrov DA, Fisher DS, Sherlock G. 2015. Quantitative evolutionary dynamics using high-resolution lineage tracking. Nature. 519(7542):181–186. doi:10.1038/nature14279. 25731169 PMC4426284

[jkaf081-B40] Lewontin RC . 1986. How important is genetics for an understanding of evolution? Am Zoöl. 26(3):811–820. doi:10.1093/icb/26.3.811.

[jkaf081-B41] Li F, Salit ML, Levy SF. 2018. Unbiased fitness estimation of pooled barcode or amplicon sequencing studies. Cell Syst. 7(5):521–525.e4. doi:10.1016/j.cels.2018.09.004. 30391162 PMC6265064

[jkaf081-B42] Mallet J . 1989. The evolution of insecticide resistance: have the insects won? Trends Ecol Evol. 4(11):336–340. doi:10.1016/0169-5347(89)90088-8. 21227375

[jkaf081-B43] Murray RL, Cutter AD. 2011. Experimental evolution of sperm count in protandrous self-fertilizing hermaphrodites. J Exp Biol. 214(10):1740–1747. doi:10.1242/jeb.053181. 21525321

[jkaf081-B44] Prichard RK . 2007. Ivermectin resistance and overview of the consortium for anthelmintic resistance SNPs. Expert Opin Drug Discov. 2(Supp1):S41–S52. doi:10.1517/17460441.2.s1.s41. 23489032

[jkaf081-B45] Pu J, Chung H. 2024. New and emerging mechanisms of insecticide resistance. Curr Opin Insect Sci. 63:101184. doi:10.1016/j.cois.2024.101184. 38458436

[jkaf081-B46] R Core Team . 2023. R: A Language and Environment for Statistical Computing. Vienna, Austria: R Foundation for Statistical Computing. https://www.R-project.org/.

[jkaf081-B47] Roff . 1992. The Evolution of Life Histories. New York: Chapman and Hall.

[jkaf081-B48] Rudman SM, Greenblum SI, Rajpurohit S, Betancourt NJ, Hanna J, Tilk S, Yokoyama T, Petrov DA, Schmidt P. 2022. Direct observation of adaptive tracking on ecological time scales in *Drosophila*. Science. 375(6586):eabj7484. doi:10.1126/science.abj7484. 35298245 PMC10684103

[jkaf081-B49] Schmidlin K, Apodaca S, Newell D, Sastokas A, Kinsler G, Geiler-Samerotte K. 2024. Distinguishing mutants that resist drugs via different mechanisms by examining fitness tradeoffs. eLife. 13:RP94144 . doi:10.7554/eLife.94144.1. 39255191 PMC11386965

[jkaf081-B50] Sharon E, Chen SA, Khosla NM, Smith JD, Pritchard JK, Fraser HB. 2018. Functional genetic variants revealed by massively parallel precise genome editing. Cell. 175(2):544–557.e16. doi:10.1016/j.cell.2018.08.057. 30245013 PMC6563827

[jkaf081-B51] Shaver AO, Miller IR, Schaye ES, Moya ND, Collins JB, Wit J, Blanco AH, Shao FM, Andersen EJ, Khan SA, et al 2024. Quantifying the fitness effects of resistance alleles with and without anthelmintic selection pressure using *Caenorhabditis elegans*. PLOS Pathog. 20(5):e1012245. doi:10.1371/journal.ppat.1012245. 38768235 PMC11142691

[jkaf081-B52] Shoop WL . 1993. Ivermectin resistance. Parasitol Today. 9(5):154–159. doi:10.1016/0169-4758(93)90136-4. 15463742

[jkaf081-B53] Stevenson ZC, Moerdyk-Schauwecker MJ, Banse SA, Patel DS, Lu H, Phillips PC. 2023. High-throughput library transgenesis in *Caenorhabditis elegans* via transgenic arrays resulting in diversity of integrated sequences (TARDIS). eLife. 12:RP84831. doi:10.7554/elife.84831.3. 37401921 PMC10328503

[jkaf081-B54] Su H, Dent J. 2015. Mutation of the *glc-2* gene may confer dominant ivermectin resistance. McGill Sci Undergrad Res J. 10(1):21–25. doi:10.26443/msurj.v10i1.118.

[jkaf081-B55] Sulik M, Antoszczak M, Huczyński A, Steverding D. 2023. Antiparasitic activity of ivermectin: four decades of research into a “wonder drug.”. Eur J Med Chem. 261:115838. doi:10.1016/j.ejmech.2023.115838. 37793327

[jkaf081-B56] Teotónio H, Estes S, Phillips PC, Baer CF. 2017. Experimental evolution with *Caenorhabditis* nematodes. Genetics. 206(2):691–716. doi:10.1534/genetics.115.186288. 28592504 PMC5499180

[jkaf081-B57] Tuersong W, Zhou C, Wu S, Qin P, Wang C, Di W, Liu L, Liu H, Hu M. 2022. Comparative analysis on transcriptomics of ivermectin resistant and susceptible strains of *Haemonchus contortus*. Parasit Vectors. 15(1):159. doi:10.1186/s13071-022-05274-y. 35524281 PMC9077910

[jkaf081-B58] Wakely J . 2008. Coalescent Theory: An Introduction. Greenwood Village (CO): Roberts & Company.

[jkaf081-B59] Wang J, Atolia E, Hua B, Savir Y, Escalante-Chong R, Springer M. 2015. Natural variation in preparation for nutrient depletion reveals a cost–benefit tradeoff. PLoS Biol. 13(1):e1002041. doi:10.1371/journal.pbio.1002041. 25626068 PMC4308108

[jkaf081-B60] Webster AK, Chitrakar R, Powell M, Chen J, Fisher K, Tanny RE, Stevens L, Evans K, Wei A, Antoshechkin I, et al 2022. Using population selection and sequencing to characterize natural variation of starvation resistance in *Caenorhabditis elegans*. eLife. 11:e80204. doi:10.7554/elife.80204. 35727141 PMC9262388

[jkaf081-B61] Weeks JC, Robinson KJ, Lockery SR, Roberts WM. 2018. Anthelmintic drug actions in resistant and susceptible *C. elegans* revealed by electrophysiological recordings in a multichannel microfluidic device. Int J Parasitol Drugs Drug Resist. 8(3):607–628. doi:10.1016/j.ijpddr.2018.10.003. 30503202 PMC6287544

[jkaf081-B62] Wickham H. 2016. ggplot2, Elegant Graphics for Data Analysis. 2nd ed. Cham, Switzerland: Springer. doi:10.1007/978-3-319-24277-4.

[jkaf081-B63] Zamanian M, Andersen EC. 2016. Prospects and challenges of CRISPR/Cas genome editing for the study and control of neglected vector-borne nematode diseases. FEBS J. 283(17):3204–3221. doi:10.1111/febs.13781. 27300487 PMC5053252

[jkaf081-B64] Zhang J . 2023. Patterns and evolutionary consequences of pleiotropy. Annu Rev Ecol Evol Syst. 54(1):1–19. doi:10.1146/annurev-ecolsys-022323-083451. 39473988 PMC11521367

