# Supplementary material for: Precise lineage tracking using molecular barcodes demonstrates fitness trade-offs for ivermectin resistance in nematodes
Source: G3 (Bethesda). 2025 Apr 10;15(6):jkaf081. doi: 10.1093/g3journal/jkaf081 (PMC12135011; doi:10.1093/g3journal/jkaf081)
Supplement: jkaf081_Supplementary_Data [file jkaf081_supplementary_data.zip › Supplemental_Legends_G3-2025-405750.docx]

**Supplemental Legends**

**Supplementary Data File 1.** Count Data For Fitness Development Census and Index Associated for Demultiplexing

**Supplementary Data File 2.** Fitness Analysis

**Supplementary Data File 3.** Developmental Analysis

**Supplementary Data File 4.** Key Reagents

**Figure S1.** Photograph of the liquid culture environment in our temperature-controlled unit.

**Figure S2.** Peak census size for each replicate population plotted at log_10_. Colors indicate unique replicates within a concentration. Peak population sizes often surpass a million individuals.

**Figure S3.** Developmental delay at 96 hours while developing in ivermectin. Wildtype continues to develop slowly in deleterious concentrations of ivermectin, however, it is progressing in 3nM and 4nM. At 5nM, the wildtype background remains highly stunted with very few individuals reaching adulthood.
