# Supplementary figures and images for: Precise lineage tracking using molecular barcodes demonstrates fitness trade-offs for ivermectin resistance in nematodes
Source: G3 (Bethesda). 2025 Apr 10;15(6):jkaf081. doi: 10.1093/g3journal/jkaf081 (PMC12135011; doi:10.1093/g3journal/jkaf081)

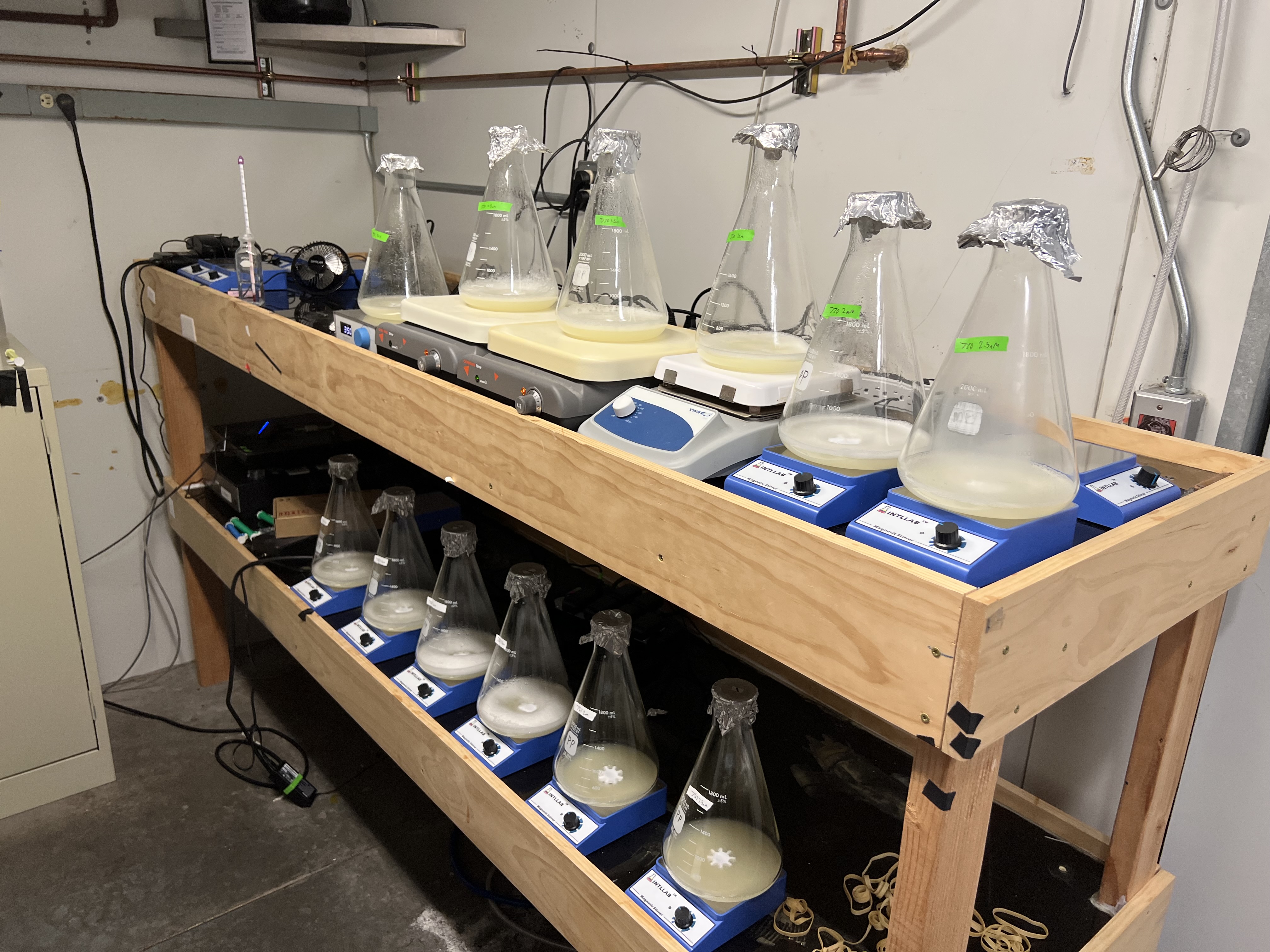

Supplement: jkaf081_Supplementary_Data [file jkaf081_supplementary_data.zip › Figure_S1_G3-2025-405750.jpg]

0nM

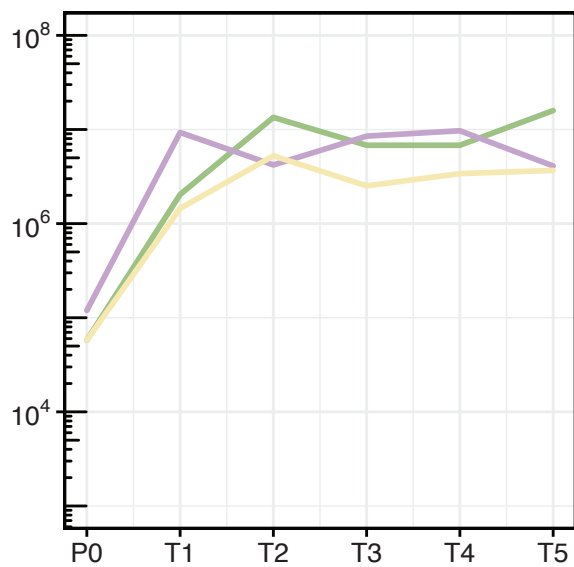

1nM

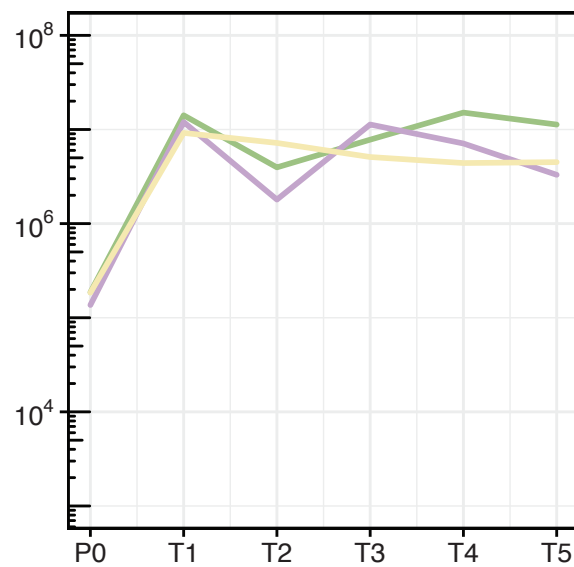

2nM

Peak Census Size

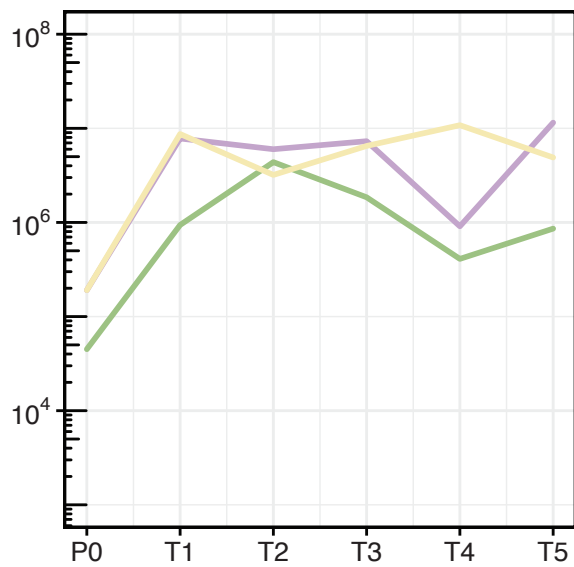

3nM

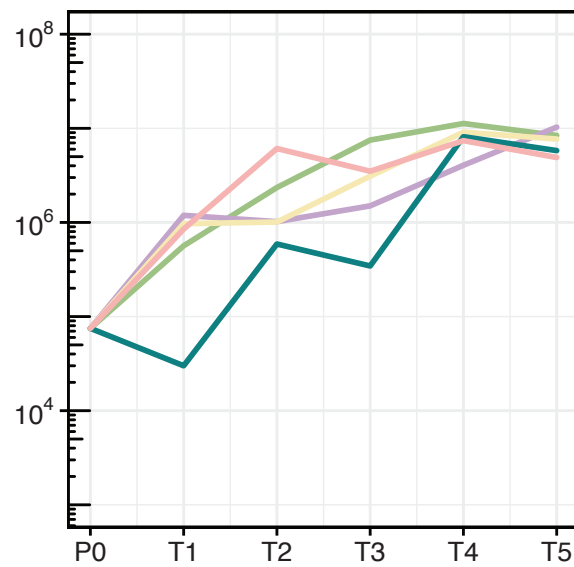

4nM

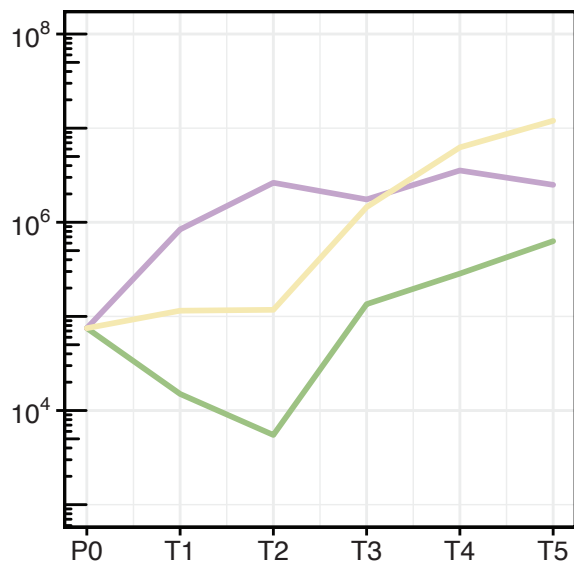

5nM

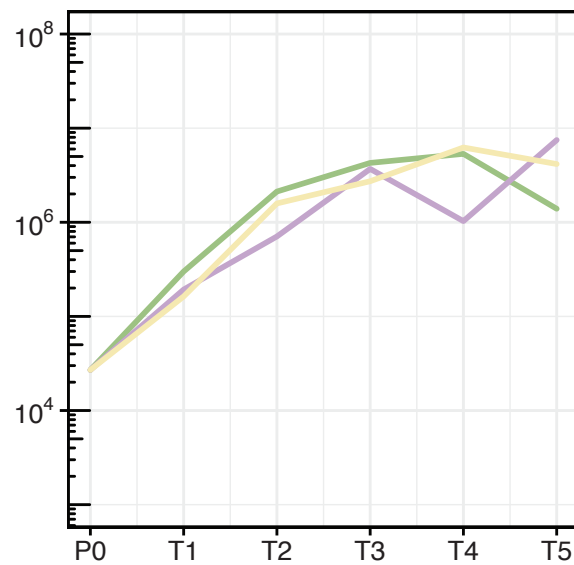

Transfers

Supplement: jkaf081_Supplementary_Data [file jkaf081_supplementary_data.zip › Figure_S2_G3-2025-405750.pdf]

Percent Adult

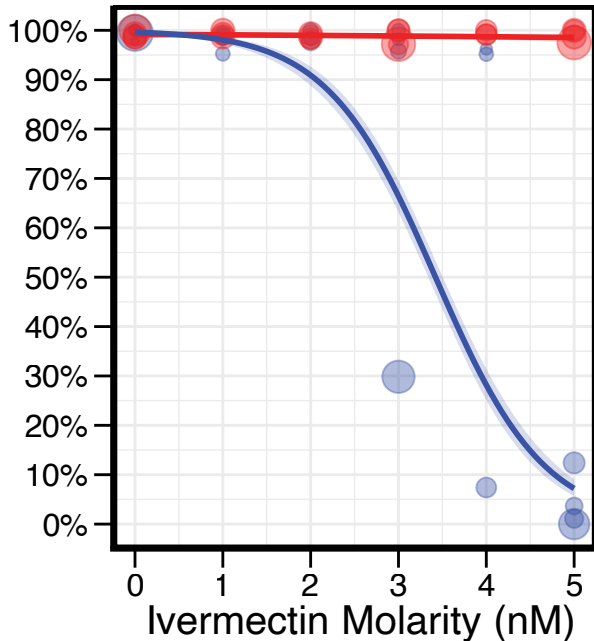

Replicate Size

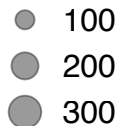

Genotype

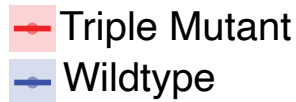

Supplement: jkaf081_Supplementary_Data [file jkaf081_supplementary_data.zip › Figure_S3_G3-2025-405750.pdf]
